# Routine Laboratory Parameters in the Differentiation of Spinal Cord Infarction and Seronegative Acute Myelitis: A Retrospective Study

**DOI:** 10.3390/jcm15187083

**Published:** 2026-09-12

**Authors:** Song Han, Mingjing Yu, Ruonan Zhang, Ling Xin, Yu Qiao, Tao Yan

**Affiliations:** Department of Neurology, Tianjin Medical University General Hospital, Tianjin 300052, China; hansong2021@tmu.edu.cn (S.H.); ymjaa369@163.com (M.Y.); zhangruonan1003@163.com (R.Z.); xinling_0515@163.com (L.X.); qiaoyu0615@163.com (Y.Q.)

**Keywords:** spinal cord infarction, differential diagnosis, acute myelitis, laboratory biomarkers

## Abstract

**Background/Objectives:** Overlapping clinical and imaging features complicate the early differentiation between spontaneous spinal cord infarction (SCI) and seronegative acute myelitis (SAM). We aimed to compare the clinical and laboratory characteristics of SCI and SAM and, as an exploratory analysis, to develop and internally validate multivariable diagnostic models evaluating whether routinely available laboratory parameters provide additional discriminatory information beyond selected clinical features. **Methods:** We retrospectively analyzed 75 patients (34 SCI, 41 SAM) treated between January 2017 and June 2025. Between-group laboratory comparisons were adjusted for multiple testing using the Benjamini–Hochberg false-discovery-rate procedure. Penalized logistic regression analyses were performed using clinical variables, laboratory variables, and their combination, with candidate variables defined on clinical and data-quality grounds rather than by univariate statistical significance. Model discrimination was evaluated using repeated nested cross-validation. Bootstrap optimism correction was additionally performed for the full-data combined model, and decision curve analysis was conducted as an exploratory secondary analysis. **Results:** SCI patients were older, more often male, and had higher prevalence of hypertension and diabetes. Radicular pain was markedly more common in SCI. After Benjamini–Hochberg correction, five laboratory parameters remained significantly different between groups: CRP, triglycerides, monocyte percentage, and absolute monocyte count were higher in SCI, whereas HDL cholesterol was lower. In the combined penalized analysis, age, hypertension, radicular pain, CRP, triglycerides, absolute monocyte count, and absolute eosinophil count were retained. The clinical, laboratory, and combined models yielded AUCs of 0.821 (95% CI 0.718–0.910), 0.756 (95% CI 0.633–0.865), and 0.871 (95% CI 0.776–0.947), respectively. Compared with the clinical model, the combined model showed a modest increase in discrimination (ΔAUC 0.050, 95% CI 0.005–0.101, *p* = 0.030). Bootstrap internal validation of the full-data combined model yielded an optimism-corrected AUC of 0.902, compared with an apparent AUC of 0.937, with a mean optimism of 0.035. Exploratory decision curve analysis showed a potential net benefit of the combined model across a range of threshold probabilities. **Conclusions:** Spontaneous SCI and SAM showed distinct clinical and laboratory profiles in this retrospective cohort. Routine laboratory parameters provided modest additional discriminatory information beyond clinical features, and the combined model maintained discrimination during internal validation. These findings are exploratory and require validation in larger, independent cohorts before clinical application.

## 1. Introduction

Ischemic disorders occupy a central position among human diseases. Owing to their abrupt onset and the potential to cause rapid and often irreversible tissue injury, they represent a major source of morbidity and disability [1]. The central nervous system comprises both the brain and the spinal cord. While cerebral ischemic diseases have been extensively investigated, research on spinal cord ischemia remains comparatively limited [2], and standardized treatment guidelines are still lacking.

Compared with cerebral infarction, the causes of acute spinal cord ischemia are more heterogeneous, partly due to the unique vascular anatomy and anatomical location of the spinal cord [3]. Previous studies have addressed secondary spinal cord infarction in certain contexts [4], but spontaneous spinal cord infarction (SCI) has received far less attention. Its underlying etiologies, pathophysiological mechanisms, and patterns of injury remain incompletely understood.

In clinical practice, spontaneous SCI is frequently misdiagnosed. Clinically, affected patients may develop acute weakness, sensory loss, back or radicular pain, and bowel or bladder dysfunction. Several factors may contribute to this. First, its clinical presentation and magnetic resonance imaging (MRI) findings often overlap with those of other, more common causes of acute myelopathy [5]. Second, SCI is relatively rare [6], which may lower clinical suspicion at initial evaluation. Third, current diagnostic approaches rely predominantly on imaging, yet MRI findings can be atypical or even falsely negative, particularly in the early stage [7]. Treatment is generally directed at the underlying vascular cause and commonly includes antiplatelet therapy, vascular risk-factor management, and rehabilitation, although controlled evidence for specific acute interventions remains limited [8,9].

Seronegative acute myelitis (SAM) is an inflammatory spinal cord syndrome characterized by motor weakness, sensory disturbance, and autonomic dysfunction that usually evolves over hours to days. MRI commonly demonstrates intramedullary T2-hyperintense lesions, while cerebrospinal fluid (CSF) pleocytosis, elevated protein, or intrathecal immunoglobulin synthesis may support an inflammatory process. Diagnosis requires exclusion of compressive, vascular, infectious, metabolic, neoplastic, paraneoplastic, and systemic autoimmune causes. Testing for aquaporin-4 immunoglobulin G (AQP4-IgG), myelin oligodendrocyte glycoprotein immunoglobulin G (MOG-IgG), and glial fibrillary acidic protein immunoglobulin G (GFAP-IgG) helps identify antibody-associated disorders. In this study, SAM refers to cases negative for these antibodies after relevant alternative diagnoses were excluded. Although controlled evidence is limited, acute immunotherapy commonly begins with high-dose corticosteroids; plasma exchange or intravenous immunoglobulin may be considered in severe or steroid-refractory cases, together with rehabilitation and etiology-specific follow-up [10,11,12].

Accurate differential diagnosis is therefore essential. In our clinical experience, distinguishing acute spontaneous SCI from seronegative acute myelitis (SAM) can be particularly challenging. Both conditions are relatively uncommon, and in many cases the precise etiology remains unclear—especially when disease-specific antibodies such as AQP4, MOG, or GFAP are absent. Both conditions may cause acute motor, sensory, and sphincter dysfunction and may produce overlapping spinal cord T2-hyperintense lesions. A hyperacute course, acute pain, vascular risk factors, diffusion restriction, and vertebral body infarction favor SCI, whereas a more subacute evolution and inflammatory CSF findings favor SAM; however, no single feature is diagnostic. Identifying objective and widely accessible biomarkers that can help differentiate SCI from inflammatory causes would facilitate earlier and more accurate diagnosis, and may help avoid unnecessary or potentially harmful treatments. Accordingly, we compared clinical characteristics and routinely measured laboratory parameters between spontaneous SCI and SAM and, as an exploratory analysis, developed and internally validated clinical, laboratory, and combined multivariable diagnostic models to evaluate whether routinely available laboratory parameters could provide additional discriminatory information beyond selected clinical features.

## 2. Materials and Methods

### 2.1. Study Design and Participants

We conducted a retrospective analysis of patients diagnosed with acute spontaneous spinal cord infarction or acute myelitis at the Department of Neurology, Tianjin Medical University General Hospital, between January 2017 and June 2025. The diagnosis of spinal cord infarction was established by the presence of acute neurological deficits attributable to the spinal cord, with symptom onset within 72 h, and neuroimaging (MRI) findings consistent with ischemic injury, after exclusion of other etiologies. Seronegative acute myelitis was defined according to the diagnostic and exclusion criteria detailed below. Two experienced neurologists and a neuroradiologist independently reviewed all cases, and any discrepancies were resolved by consensus. The patient selection process is summarized in Supplementary Figure S1.

Available MRI characteristics, including imaging timing, lesion location and length, initial MRI findings, DWI findings, vertebral body signal abnormalities, and anterior spinal artery territory patterns, are summarized in Supplementary Table S1. Short-term motor improvement was defined as an increase of at least one grade on the Medical Research Council muscle strength scale between admission and discharge.

### 2.2. Definition and Ascertainment of Acute Myelitis

Seventy potential AM cases were retrospectively reassessed according to the 2002 Transverse Myelitis Consortium Working Group criteria, together with available follow-up information; 29 were excluded, leaving 41 patients. Alternative compressive, vascular, infectious, metabolic, neoplastic, paraneoplastic, and systemic autoimmune causes were excluded. All included patients were negative for serum AQP4-IgG, MOG-IgG, and GFAP-IgG; AQP4-IgG and MOG-IgG were tested using cell-based assays. NMOSD and MOGAD were also excluded. CSF cell count, protein, and oligoclonal bands were available in 40 patients, and the IgG index in six. Follow-up data were available for 30 patients, none of whom was subsequently reclassified.

### 2.3. Definition and Ascertainment of Spinal Cord Infarction

Seventy-nine patients with a potential diagnosis of spontaneous spinal cord infarction (SCI) were initially screened. SCI was diagnosed on the basis of an acute neurological syndrome attributable to the spinal cord, symptom onset within 72 h, imaging findings compatible with spinal cord ischemia on either the initial or subsequent examination, and exclusion of alternative compressive, inflammatory, infectious, metabolic, neoplastic, and other causes of acute myelopathy. An initially negative MRI did not exclude SCI. Among the 33 SCI patients with available initial MRI data, 15 had initially negative examinations; all 15 subsequently demonstrated imaging findings considered supportive of spinal cord ischemia on subsequent imaging evaluation. Patients admitted more than seven days after symptom onset and those whose diagnosis was subsequently revised to another disorder during follow-up were excluded. After these exclusions, 34 patients with spontaneous SCI were included in the analysis. The patient-selection process is shown in Supplementary Figure S1. For the purposes of this study, spontaneous SCI was defined as spinal cord infarction occurring in the absence of a temporally related aortic surgical or endovascular intervention, spinal surgery or other spinal procedure, major trauma, or documented sustained systemic hypotension, shock, or circulatory arrest. Patients whose SCI was attributable to any of these secondary or periprocedural causes were excluded.

### 2.4. Data Collection

Routine blood samples were collected after an overnight fast on the morning following admission. Laboratory measurements included immunologic, inflammatory, coagulation, cardiac, lipid, hepatic, renal, and hematologic parameters. Because of the retrospective design, the timing of blood sampling relative to acute treatments, including corticosteroids, intravenous immunoglobulin, antithrombotic therapy, and intravenous fluids, could not be determined consistently for all patients.

Demographic characteristics, vascular risk factors (hypertension, diabetes mellitus, smoking, alcohol consumption, atrial fibrillation), clinical features (presence of radicular pain, precipitating events), and treatment modalities were extracted from medical records. Radicular pain was treated as a binary clinical feature based on its documented presence or absence in the admission record. Laboratory parameters including complement C3 and C4, immunoglobulins (IgG, IgA, IgM), inflammatory markers (C-reactive protein [CRP], high-sensitivity CRP), coagulation markers (fibrinogen, D-dimer, thrombin time, international normalized ratio [INR]), cardiac biomarkers (B-type natriuretic peptide [BNP], troponin T [TnT]), lipid profile (total cholesterol, triglycerides, high-density lipoprotein cholesterol [HDL-C], low-density lipoprotein cholesterol), liver and renal function tests (alanine aminotransferase, aspartate aminotransferase, creatinine, total protein, albumin), creatine kinase (CK) and its isoenzyme (CK-MB), complete blood counts (white blood cell count, differential counts, platelet count), and cerebrospinal fluid (CSF) parameters (opening pressure, protein, lactate dehydrogenase, white blood cell count) were collected from laboratory records. All laboratory tests were performed using standard clinical methods within 48 h of admission.

A total of 39 blood laboratory parameters were analyzed, as detailed in Supplementary Table S3. S100 was included among these parameters. CSF measurements were used only as supportive diagnostic information and were not included in the 39 laboratory comparisons, multiple-testing correction, or prediction models.

### 2.5. Statistical Analysis

Continuous demographic and clinical variables were assessed for normality using the Shapiro–Wilk test. Normally distributed variables are presented as mean ± standard deviation and were compared using the independent-samples *t* test, whereas non-normally distributed variables are presented as median (interquartile range) and were compared using the two-sided Mann–Whitney U test. Categorical variables are presented as number (percentage) and were compared using the chi-square test or Fisher’s exact test, as appropriate.

Because the laboratory variables generally showed skewed distributions and the group sizes were modest, all 39 laboratory parameters were summarized as median (interquartile range) and compared between the SCI and SAM groups using two-sided Mann–Whitney U tests, irrespective of the individual normality-test results. Raw *p* values from these 39 comparisons were adjusted using the Benjamini–Hochberg false-discovery-rate procedure. A two-sided *p* value < 0.05 was considered nominally significant, and a Benjamini–Hochberg-adjusted q value < 0.05 was considered statistically significant after correction for multiple comparisons. Rank-biserial correlations were calculated as effect-size estimates, with positive values indicating higher measurements in the SCI group.

To address potential multicollinearity, we performed least absolute shrinkage and selection operator (LASSO) logistic regression for three predictor sets defined for the revised analysis. SCI was coded as 1 and SAM as 0. The clinical candidate set comprised age, sex, hypertension, diabetes, and radicular pain. The laboratory candidate set comprised IgM, CRP, fibrinogen, triglycerides, high-density lipoprotein cholesterol, creatinine, absolute monocyte count, and absolute eosinophil count. These variables were selected to provide a limited set of routinely available and clinically interpretable measures spanning immune/inflammatory activity (IgM and CRP), coagulation (fibrinogen), vascular-metabolic status (triglycerides and HDL-C), renal/vascular comorbidity (creatinine), and circulating leukocyte profiles (absolute monocyte and eosinophil counts). To reduce redundancy, representative variables were chosen within related laboratory domains, and absolute leukocyte counts were preferred over the corresponding percentages for candidate modeling. The combined candidate set included all 13 clinical and laboratory variables. S100 and B-type natriuretic peptide were not considered as candidate predictors because 88.0% and 24.0% of their values were missing, respectively.

Before imputation, the number and proportion of missing observations were quantified for each clinical and laboratory variable, both overall and separately in the SCI and SAM groups (Supplementary Table S3). Within each model-development or validation training set, missing predictor values were replaced with the corresponding training-set medians, and predictors were standardized using the training-set means and standard deviations. The resulting imputation and standardization parameters were then applied unchanged to the corresponding held-out data. Within each training set, the penalty parameter was selected using 10-fold cross-validation and the one-standard-error criterion (λ_1_SE). All preprocessing, penalty selection, variable selection, and model fitting steps were repeated within each outer cross-validation fold and bootstrap resample to prevent information leakage. After completion of internal validation, the combined LASSO model was refitted using the complete dataset with the same preprocessing and tuning procedure to provide the final model specification.

Discriminative ability was assessed using the area under the receiver operating characteristic curve (AUC) based on aggregated out-of-fold predictions generated by 50 repetitions of nested stratified five-fold cross-validation. In each repetition, preprocessing, penalty selection, and model fitting were confined to the outer training folds, and predictions were generated for the held-out fold. The 50 out-of-fold predictions for each participant were averaged before performance estimation. Ninety-five percent confidence intervals for the AUCs were obtained using 10,000 patient-level bootstrap resamples. Differences in AUC between models were evaluated using paired patient-level bootstrap resampling of the corresponding out-of-fold predictions. *p* values testing each AUC against 0.5 were calculated using the DeLong method. To evaluate potential overfitting, bootstrap internal validation with 1000 resamples was performed for the final combined model. In every bootstrap resample, the complete model-development procedure was repeated, including median imputation, standardization, inner 10-fold cross-validation, selection of lambda.1se, variable selection, and penalized model fitting. Duplicate copies of the same participant were assigned to the same inner fold. Each refitted model was evaluated in both the bootstrap sample and the original cohort. Mean optimism was subtracted from the apparent AUC; the Brier score was corrected in the corresponding direction. Predictor selection frequency was calculated as the proportion of bootstrap refits in which the predictor had a nonzero coefficient. Decision curve analysis (DCA) was conducted to assess the clinical net benefit of the combined model across a range of threshold probabilities, with net benefit compared against the “treat all” and “treat none” strategies. In this diagnostic context, “treat all” and “treat none” are conventional DCA comparator strategies and do not represent literal treatment recommendations. Net benefit was corrected pointwise for optimism using the same 1000 full-procedure bootstrap refits over threshold probabilities from 0.01 to 0.80. Because no specific clinical intervention or decision threshold was prespecified, DCA was considered an exploratory analysis. Calibration of the full-data combined model was assessed using the calibration intercept, calibration slope, Brier score, and a smoothed calibration curve. Apparent calibration statistics were calculated from predictions generated by the model fitted to the complete dataset. Internal validation was performed using 1000 bootstrap samples, with the complete model-development procedure, including median imputation, predictor standardization, penalty selection, and model fitting, repeated within each bootstrap sample. Optimism in the Brier score and the smoothed calibration curve was estimated by comparing performance in each bootstrap sample with performance of the corresponding bootstrap-derived model in the original cohort. The mean optimism was then used to obtain optimism-corrected estimates. Scalar optimism correction of the calibration slope was numerically unstable because several bootstrap refits produced nearly constant predictions and extremely large training-sample calibration slopes. Therefore, the calibration slope is reported as an apparent estimate, whereas the Brier score and calibration curve are presented as optimism-corrected results.

For analyses other than the FDR-adjusted laboratory comparisons, two-sided *p* < 0.05 was considered statistically significant. Analyses were performed using SPSS (version 26.0, IBM Corp., Armonk, NY, USA) and R (version 4.5.1, R Foundation for Statistical Computing, Vienna, Austria) with the packages rms(8.1-1), glmnet(4.1-9), pROC(1.19.0.1) and rmda(1.6).

## 3. Results

### 3.1. Baseline Characteristics

A total of 75 patients were included, comprising 41 with acute myelitis and 34 with spinal cord infarction. Baseline characteristics are summarized in Table 1. Patients with infarction were significantly older (62.85 ± 12.73 vs. 47.29 ± 17.48 years, *p* < 0.001) and more often male (79.4% vs. 48.8%, *p* = 0.006). Hypertension (61.8% vs. 17.1%, *p* < 0.001) and diabetes (41.2% vs. 12.2%, *p* = 0.004) were more prevalent in the infarction group. No significant differences were observed in smoking, alcohol consumption, or atrial fibrillation (all *p* > 0.05).

Radicular pain was reported by 61.8% of infarction patients compared to only 4.9% of myelitis patients (*p* < 0.001). Precipitating events also differed significantly (*p* < 0.001): myelitis was more often preceded by infection (39.0%) or had no identifiable trigger (51.2%), whereas infarction was frequently associated with physical activity (32.4%) or had no clear trigger (41.2%).

### 3.2. Laboratory Characteristics

Routine laboratory parameters were compared between the SCI and SAM groups (Table 2). At the nominal significance level, patients with SCI had higher levels of CRP, fibrinogen, BNP, troponin T, triglycerides, creatinine, monocyte percentage, eosinophil percentage, absolute monocyte count, and absolute eosinophil count, whereas IgM, HDL cholesterol, and albumin levels were lower than those in patients with SAM (all unadjusted *p* < 0.05).

After adjustment for multiple comparisons using the Benjamini–Hochberg procedure across all 39 laboratory parameters, five differences remained statistically significant. Compared with the SAM group, patients with SCI had higher CRP levels (0.84 [0.49–3.34] vs. 0.30 [0.17–0.45] mg/L; q < 0.001), triglyceride levels (1.65 [1.34–2.40] vs. 1.18 [0.85–1.62] mmol/L; q = 0.025), monocyte percentages (7.70 [6.83–9.43]% vs. 6.45 [5.63–7.50]%; q = 0.022), and absolute monocyte counts (0.62 [0.49–0.78] vs. 0.45 [0.36–0.62] × 10^9^/L; q = 0.044), whereas HDL cholesterol levels were lower (0.90 [0.81–1.07] vs. 1.08 [0.97–1.26] mmol/L; q = 0.049). The corresponding rank-biserial correlations ranged from |0.385| to |0.614|, with the largest between-group difference observed for CRP.

### 3.3. Predictor Selection, Selection Stability, and Model Discrimination

**Figure 1:**
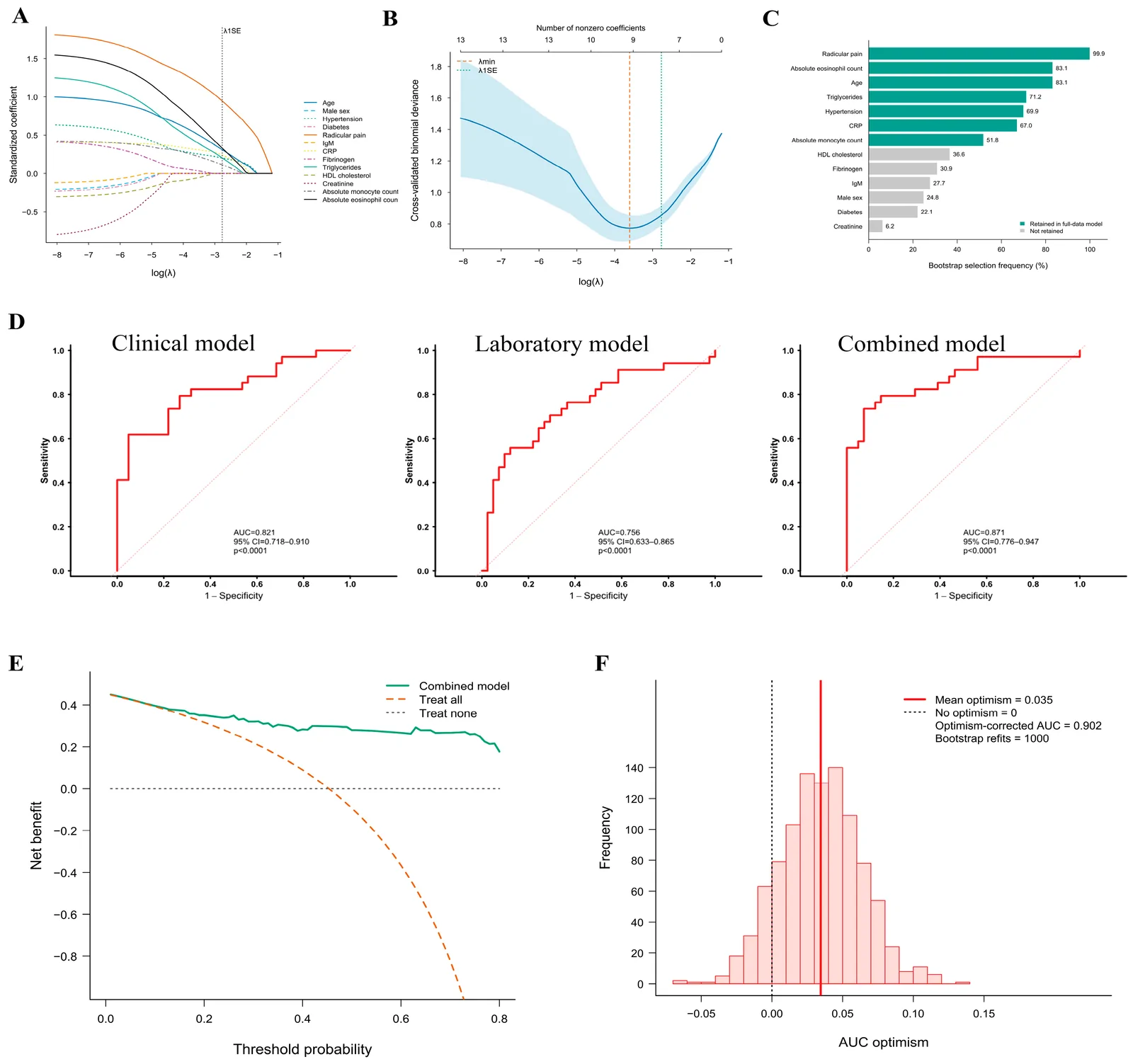
Predictor selection, selection stability, and discriminative performance of the clinical, laboratory, and combined models. (**A**) Coefficient profiles of the candidate predictors across different values of the regularization parameter [log(λ)] in the combined model. The vertical dotted line indicates the penalty parameter selected using the one-standard-error criterion (λ_1_SE). (**B**) Cross-validation curve for penalty-parameter selection. The solid line and shaded area represent the mean cross-validated deviance and its standard error, respectively. The dashed vertical lines indicate λmin and λ_1_SE, and the upper axis shows the corresponding number of nonzero coefficients. (**C**) Bootstrap selection frequencies of the candidate predictors based on 1000 repetitions of the complete model-development procedure. Teal bars indicate predictors retained in the final model fitted to the complete dataset, whereas gray bars indicate predictors not retained. (**D**) Receiver operating characteristic curves for the clinical, laboratory, and combined models based on out-of-fold predictions from repeated nested five-fold cross-validation. (**E**) Optimism-corrected decision curve analysis of the combined model. (**F**) Distribution of AUC optimism across 1000 bootstrap resamples in which the complete model-development procedure was repeated.

Using the one-standard-error criterion, the clinical model retained age, hypertension, and radicular pain, whereas the laboratory model retained C-reactive protein, triglycerides, absolute monocyte count, and absolute eosinophil count. The combined model retained all seven of these predictors (Figure 1A,B). Bootstrap analysis showed that radicular pain had the highest selection frequency (99.9%), followed by age and absolute eosinophil count (both 83.1%), triglycerides (71.2%), hypertension (69.9%), C-reactive protein (67.0%), and absolute monocyte count (51.8%). All other candidate predictors were selected in fewer than 50% of bootstrap samples (Figure 1C).

Based on aggregated out-of-fold predictions from repeated nested five-fold cross-validation, the clinical, laboratory, and combined models yielded AUCs of 0.821 (95% CI, 0.718–0.910), 0.756 (95% CI, 0.633–0.865), and 0.871 (95% CI, 0.776–0.947), respectively (Figure 1D and Table 3). Compared with the clinical model, the combined model showed a modest improvement in discrimination (ΔAUC, 0.050; 95% CI, 0.005–0.101; *p* = 0.030). The combined model also showed greater discrimination than the laboratory model (ΔAUC, 0.115; 95% CI, 0.005–0.232; *p* = 0.040), whereas the laboratory and clinical models did not differ significantly (ΔAUC, −0.065; 95% CI, −0.205 to 0.074; *p* = 0.362).

### 3.4. Model Specification

In the full-data combined LASSO fit, the penalty parameter selected using the one-standard-error criterion was 0.062875. Seven predictors had nonzero penalized coefficients: age, hypertension, radicular pain, CRP, triglycerides, absolute monocyte count, and absolute eosinophil count. On the original measurement scale, the linear predictor was calculated as follows:

LP=−2.937179+0.018072×Age+0.417311×Hypertension+2.052281×Radicular pain+0.122950×CRP+0.187146×Triglycerides+0.482027×Absolute monocyte count+2.594441×Absolute eosinophil count

Hypertension and radicular pain were coded as 1 when present and 0 when absent. CRP was expressed in mg/L, triglycerides in mmol/L, and the absolute monocyte and eosinophil counts in ×10^9^/L. The predicted probability of spinal cord infarction was calculated as P(SCI)=1/[1+exp(−LP)]. These coefficients describe the final model fitted to the complete dataset and were not optimism-corrected.

#### Internal Validation and Calibration

To assess potential overfitting, bootstrap internal validation was performed with 1000 resamples. The apparent AUC of the combined model was 0.937, with a mean optimism of 0.035, resulting in an optimism-corrected AUC of 0.902. These findings indicated some degree of optimism in the apparent discrimination.

The apparent calibration intercept and slope of the full-data combined model were 0.000 and 2.083, respectively. Bootstrap internal validation yielded an optimism-corrected calibration intercept of 0.039. A scalar optimism-corrected calibration slope was not reported because the optimism estimate was numerically unstable across bootstrap refits. The apparent Brier score was 0.106 and increased to 0.129 after optimism correction. The optimism-corrected calibration curve showed some deviation from the ideal line (Supplementary Figure S2).

### 3.5. Decision Curve Analysis

Decision curve analysis showed that the combined clinical and laboratory model provided a higher net benefit than the “treat all” and “treat none” strategies across a broad range of threshold probabilities, approximately from 15% to 80%. The separation from the “treat all” strategy became more apparent at intermediate and higher thresholds, while the net benefit remained positive throughout most of this range. Given that no specific clinical action was prespecified for these probability thresholds, the decision-curve findings should be regarded as exploratory and require evaluation in a clearly defined clinical decision context.

### 3.6. Discriminative Performance of Individual Laboratory Parameters

**Figure 2:**
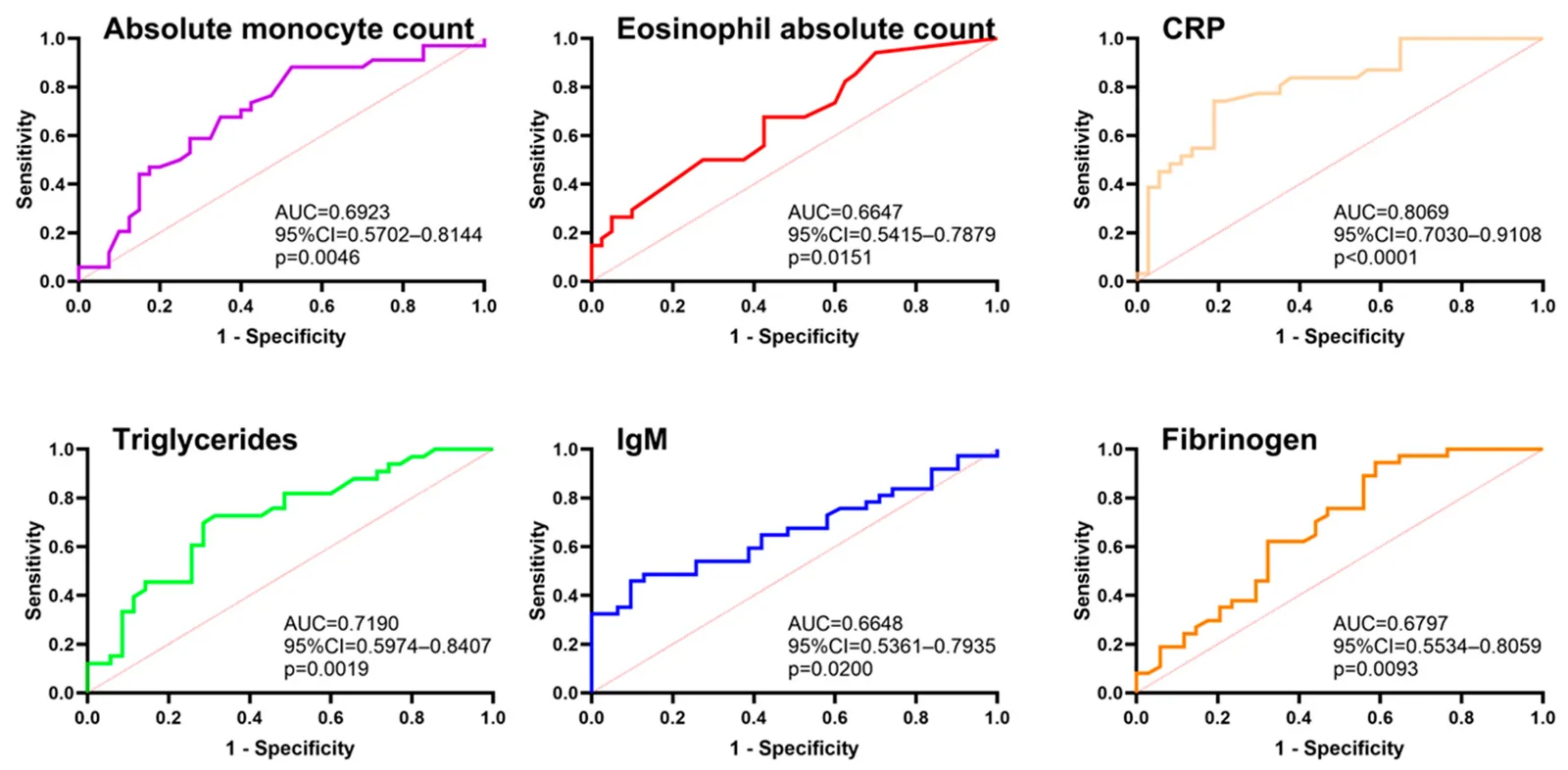
Receiver operating characteristic curves for six selected laboratory parameters. Receiver operating characteristic curves are shown for C-reactive protein (CRP), triglycerides, absolute monocyte count, absolute eosinophil count, immunoglobulin M (IgM), and fibrinogen. These single-marker analyses were performed as descriptive secondary analyses and were not used for predictor selection. Among these parameters, CRP, triglycerides, absolute monocyte count, and absolute eosinophil count were retained in the laboratory penalized model, whereas IgM and fibrinogen were not retained. The area under the receiver operating characteristic curve (AUC), 95% confidence interval (CI), and *p* value for each parameter are displayed in the corresponding panel. The diagonal reference line indicates discrimination no better than chance.

As a descriptive secondary analysis, ROC analysis was performed for six selected laboratory parameters: CRP, triglycerides, absolute monocyte count, absolute eosinophil count, IgM, and fibrinogen (Figure 2). CRP showed the highest individual discrimination, with an AUC of 0.807 (95% CI, 0.703–0.911; *p* < 0.001), followed by triglycerides (AUC, 0.719; 95% CI, 0.597–0.841; *p* = 0.002). The AUCs were 0.692 (95% CI, 0.570–0.814; *p* = 0.005) for absolute monocyte count, 0.680 (95% CI, 0.553–0.806; *p* = 0.009) for fibrinogen, 0.665 (95% CI, 0.536–0.794; *p* = 0.020) for IgM, and 0.665 (95% CI, 0.542–0.788; *p* = 0.015) for absolute eosinophil count. Of these six parameters, CRP, triglycerides, absolute monocyte count, and absolute eosinophil count were retained in the laboratory-penalized model, whereas IgM and fibrinogen were not retained.

### 3.7. Treatment Outcomes in the Infarction Subgroup

Among the 34 patients with spinal cord infarction, 12 (35.3%) showed an improvement of at least one MRC grade from admission to discharge, whereas 22 (64.7%) did not. Treatment distributions according to short-term motor improvement are shown in Supplementary Table S2. Given the small sample size, nonrandomized treatment allocation, and potential confounding by indication, these data were considered exploratory and were not used to draw conclusions regarding treatment efficacy.

## 4. Discussion

This study systematically compared clinical characteristics, laboratory profiles, and treatment outcomes between patients with acute spinal cord infarction and seronegative acute myelitis. Our findings indicate that despite overlapping clinical presentations, these two entities have distinct demographic, clinical, and laboratory features and that routinely available laboratory parameters may provide additional discriminatory information beyond clinical features.

### 4.1. Clinical Features

Consistent with the vascular nature of spinal cord infarction, affected patients were older, more frequently male, and had a higher burden of vascular risk factors such as hypertension and diabetes. The presence of radicular pain (61.8% in infarction vs. 4.9% in myelitis) emerged as a key distinguishing symptom. This may reflect acute ischemia of the spinal nerve roots or the dorsal horn, leading to neuropathic pain, which is less characteristic of inflammatory myelitis. Furthermore, infarction was often precipitated by physical activity (32.4%), suggesting a possible hemodynamic trigger, whereas myelitis was frequently preceded by infection (39.0%) consistent with an inflammatory context. These observations, however, are derived from retrospective data and should be interpreted with caution.

### 4.2. Laboratory Characteristics and Model Performance

Between-group comparisons revealed differences across multiple domains, including inflammatory (CRP, fibrinogen), cardiac (BNP, TnT), lipid (triglycerides, HDL), renal (creatinine), and immune/inflammatory cell (monocytes, eosinophils) markers. After Benjamini–Hochberg correction across 39 laboratory parameters, only five between-group differences remained: SCI was associated with higher CRP, triglyceride, monocyte percentage, and absolute monocyte count values and lower HDL-C. CRP showed the largest rank-biserial effect and the highest individual AUC (0.807). These corrected results narrow the original broad laboratory profile to a smaller set of more robust group differences within this cohort.

The penalized models addressed a different question from the between-group comparisons: which variables contributed jointly to diagnostic discrimination. The laboratory model retained CRP, triglycerides, absolute monocyte count, and absolute eosinophil count, and the combined model retained these four variables together with age, hypertension, and radicular pain. Absolute eosinophil count was retained despite not remaining significant after multiplicity correction in the marginal group comparison. Conversely, HDL-C remained different between groups after multiplicity correction but was not retained in the combined penalized model. These findings illustrate why marginal association and multivariable predictive contribution should be interpreted separately.

Repeated nested five-fold cross-validation yielded AUCs of 0.821 for the clinical model, 0.756 for the laboratory model, and 0.871 for the combined model. The combined model outperformed the clinical model (ΔAUC, 0.050; 95% CI, 0.005–0.101; *p* = 0.030), although the magnitude of improvement was modest. Given the small sample size and variability in predictor-selection frequencies, this finding should be interpreted as preliminary evidence of incremental discrimination. The modest absolute improvement also suggests that its clinical relevance may be limited in isolation and requires confirmation in larger independent cohorts. Thus, clinical characteristics accounted for much of the discriminatory information, while laboratory parameters provided complementary information rather than replacing clinical assessment.

The full-development bootstrap produced an apparent AUC of 0.937 and an optimism-corrected AUC of 0.902, with the Brier score changing from 0.106 to 0.129. The more conservative out-of-fold estimate of 0.871 is therefore the most appropriate summary of expected discrimination in similar patients. Calibration analysis showed some deviation from ideal calibration, indicating that individual predicted probabilities should be interpreted cautiously. Exploratory decision-curve analysis suggested net benefit across a range of threshold probabilities, but its clinical meaning remains uncertain because no specific clinical action was linked to these thresholds.

### 4.3. Potential Pathophysiological Implications

The laboratory parameters identified in this study reflect differences across several biological domains, including lipid metabolism, immune activity, and vascular comorbidity. However, these findings should primarily be interpreted as clinical associations and discriminatory signals rather than evidence of specific biological mechanisms. Abnormalities in triglycerides and HDL cholesterol were particularly notable [13,14]. Elevated triglycerides and reduced HDL are well-recognized components of an atherogenic lipid profile and are strongly linked to vascular disease [15,16,17,18]. In our cohort, these changes most likely reflect underlying cardiovascular risk rather than an acute reaction to spinal ischemia. This interpretation is supported by the older age and higher prevalence of hypertension and diabetes in the infarction group. The relatively higher HDL levels in myelitis patients are consistent with a lower vascular risk background. In this respect, SCI resembles cerebral ischemia in that systemic metabolic factors appear to contribute substantially to disease susceptibility. Triglycerides, but not HDL-C, were retained in the laboratory and combined models, suggesting that the two lipid measures did not provide equivalent multivariable information.

Differences in IgM levels may also reflect differences in immune background between the two groups. IgM is central to early humoral responses, and its nominally higher level in myelitis is therefore of potential biological interest. However, IgM did not remain significant after multiplicity correction and was not retained in the penalized models. The observed difference should therefore not be interpreted as evidence of enhanced humoral immune activation in seronegative myelitis [19,20]. Whether natural IgM antibodies play a modulatory role in ischemic injury remains uncertain, but this possibility merits further investigation [21,22].

The elevation of monocytes in SCI patients may reflect differences in innate immune activation between the two groups [23]. Monocytes are key mediators of vascular inflammation and post-ischemic tissue responses [24,25], and their increase may reflect systemic endothelial dysfunction or atherosclerotic activity [23]. In the present study, both monocyte percentage and absolute monocyte count remained different after multiplicity correction, and absolute monocyte count was retained in the laboratory and combined models. This convergence supports monocyte count as a useful component of the multivariable profile, while not determining whether monocytes directly mediate spinal cord injury.

The relative increase in eosinophils in SCI, although modest and generally within reference ranges, is noteworthy [26,27]. Emerging evidence indicates that eosinophils may contribute to endothelial injury and prothrombotic states [28,29,30]. However, the between-group difference in eosinophil count did not remain significant after multiplicity correction, despite its retention in the penalized model. Its relevance to spinal cord ischemia therefore remains uncertain and should be considered hypothesis-generating [31].

CRP provided the clearest inflammatory signal: it remained different after multiplicity correction, had the highest individual AUC, and was retained in the laboratory and combined models. CRP is a nonspecific acute-phase marker but is consistently linked to vascular inflammation and ischemic outcomes [32,33,34,35]. Fibrinogen showed modest individual discrimination (AUC, 0.680) but did not survive multiplicity correction or penalized selection; it may reflect coagulation and acute-phase activity without providing stable additional discriminatory information [35,36]. The sampling window prevents separation of chronic vascular inflammation from the acute response to tissue injury, but the consistent CRP findings justify further study in prospectively timed samples.

Overall, the laboratory findings may reflect differences in vascular risk, systemic inflammation, and circulating immune-cell profiles between SCI and seronegative myelitis. While biologically plausible, these patterns should be considered hypothesis-generating and do not establish disease-specific mechanisms.

### 4.4. Treatment Considerations

Treatment patterns in the SCI subgroup were explored descriptively. Given the small sample size, nonrandomized treatment allocation, and substantial potential for confounding by indication, these data do not permit meaningful inference regarding treatment efficacy. This likely reflects the small sample size, confounding by indication (sicker patients receiving more aggressive therapy), and the absence of standardized treatment protocols. Currently, management of spontaneous spinal cord infarction largely follows cerebral ischemia guidelines, but the anatomical and pathological differences between brain and spinal cord ischemia call for dedicated studies. The exploratory treatment comparisons should not be interpreted as evidence for or against treatment efficacy because treatment allocation was nonrandomized and strongly influenced by clinical severity and physician judgment.

### 4.5. Limitations

This study has several important limitations. First, its retrospective, single-center design is susceptible to selection bias and unmeasured confounding. Second, the sample size, particularly the infarction group (*n* = 34), limited our statistical power and precluded more complex multivariable modeling or robust subgroup analyses. Third, despite careful diagnostic review, misclassification remains possible. The SAM group was clinically heterogeneous, which may have introduced spectrum bias. Fourth, although laboratory samples were collected within 48 h of admission, the interval from symptom onset to blood sampling was not standardized. Moreover, the timing of blood sampling relative to acute treatments could not be determined consistently, and treatment-related changes in laboratory measurements therefore cannot be excluded. Fifth, several laboratory variables had missing observations, and median imputation does not fully account for the uncertainty associated with missing data. Parameters with substantial missingness, particularly BNP and S100, should therefore be interpreted cautiously. Sixth, although imputation, standardization, penalty selection, variable selection, and model fitting were repeated within the resampling procedures, the model has not undergone external validation. The apparent calibration slope deviated from the ideal value, and a scalar optimism-corrected slope could not be reported because its bootstrap estimate was numerically unstable. Calibration and overall model performance therefore require confirmation in a larger independent cohort. Seventh, the present analysis did not quantify the incremental value of the laboratory predictors beyond a comprehensive model incorporating standardized MRI and CSF findings. Finally, the exploratory treatment analysis was underpowered and did not adequately account for baseline disease severity, treatment selection, or other potential confounders.

## 5. Conclusions

In this retrospective single-center cohort, the combined model comprising age, hypertension, radicular pain, CRP, triglycerides, absolute monocyte count, and absolute eosinophil count achieved the highest out-of-fold discrimination for distinguishing spinal cord infarction from seronegative acute myelitis (AUC, 0.871; 95% CI, 0.776–0.947). Although statistically significant, the improvement over the clinical model was modest, suggesting that laboratory variables may provide complementary discriminatory information rather than replace clinical and imaging assessment. The clinical relevance of this incremental improvement remains uncertain. Among the six laboratory markers examined individually, CRP had the highest AUC, whereas IgM and fibrinogen were not retained in the final laboratory model. Prospective multicenter studies should validate the model, assess calibration, and determine its incremental value alongside standardized MRI and CSF evaluation.

## Figures and Tables

**Table 1:** Baseline characteristics of patients with acute myelitis and spinal cord infarction.

Variable	Acute Myelitis (*n* = 41)	Spinal Cord Infarction (*n* = 34)	*p* Value
**Age, years**	47.29 ± 17.48	62.85 ± 12.73	<0.001
**Male sex**	20 (48.8)	27 (79.4)	0.006
**Hypertension**	7 (17.1)	21 (61.8)	<0.001
**Diabetes**	5 (12.2)	14 (41.2)	0.004
**Smoking**	11 (26.8)	13 (38.2)	0.292
**Alcohol consumption**	9 (22.0)	12 (35.3)	0.200
**Atrial fibrillation**	1 (2.4)	3 (8.8)	0.323
**Radicular pain**	2 (4.9)	21 (61.8)	<0.001
**Precipitating event**			<0.001
- **None**	21 (51.2)	14 (41.2)	
- **Atrial fibrillation**	0 (0.0)	4 (11.8)	
- **During/after physical activity**	2 (4.9)	11 (32.4)	
- **After waking up**	1 (2.4)	3 (8.8)	
- **Post-infectious**	16 (39.0)	1 (2.9)	
- **Other**	1 (2.4)	1 (2.9)	

Data are presented as mean ± standard deviation or n (%). *p* values were calculated using the independent samples *t*-test for age, and the chi-square test or Fisher’s exact test for categorical variables, as appropriate.

**Table 2:** Routine laboratory parameters with nominal between-group differences.

Parameter	SAM, Median (IQR)	SCI, Median (IQR)	*p* Value	BH-Adjusted q Value	Rank-Biserial Correlation
**CRP (mg/L)**	0.30 (0.17–0.45)	0.84 (0.49–3.34)	<0.001	<0.001	0.614
IgM (mg/dL)	101.0 (69.0–150.0)	73.9 (60.4–97.4)	0.020	0.071	−0.330
Fibrinogen (g/L)	3.00 (2.57–3.29)	3.34 (2.79–4.31)	0.009	0.052	0.359
BNP (pg/mL)	12.5 (5.6–26.9)	40.2 (9.5–176.0)	0.009	0.052	0.408
Troponin T (μg/L)	0.007 (0.004–0.011)	0.013 (0.006–0.024)	0.012	0.059	0.373
**Triglycerides (mmol/L)**	1.18 (0.85–1.62)	1.65 (1.34–2.40)	0.002	0.025	0.438
**HDL cholesterol (mmol/L)**	1.08 (0.97–1.26)	0.90 (0.81–1.07)	0.006	0.049	−0.389
Albumin (g/L)	39.0 (37.0–42.0)	37.0 (34.0–40.0)	0.039	0.118	−0.286
Creatinine (μmol/L)	55.5 (49.8–69.5)	66.0 (55.0–88.0)	0.018	0.071	0.336
**Monocyte percentage (%)**	6.45 (5.63–7.50)	7.70 (6.83–9.43)	0.001	0.022	0.442
Eosinophil percentage (%)	0.70 (0.00–1.40)	1.20 (0.53–2.00)	0.031	0.102	0.290
**Absolute monocyte count (×10^9^/L)**	0.45 (0.36–0.62)	0.62 (0.49–0.78)	0.005	0.044	0.385
Absolute eosinophil count (×10^9^/L)	0.05 (0.00–0.11)	0.10 (0.03–0.16)	0.015	0.064	0.329

Data are presented as median (interquartile range). Between-group comparisons were performed using two-sided Mann–Whitney U tests. Only parameters with an unadjusted *p* value < 0.05 are shown. *p* values for all 39 laboratory parameters were adjusted using the Benjamini–Hochberg false-discovery-rate procedure. Positive rank-biserial correlations indicate higher values in the spinal cord infarction group. After correction, CRP, monocyte percentage, triglycerides, absolute monocyte count, and HDL cholesterol remained statistically significant at q < 0.05.

**Table 3:** Discriminative Performance of the Clinical, Laboratory, and Combined Models.

**A. Model discrimination**		
**Model**	**AUC (95% CI)**	
**Clinical model**	0.821 (0.718–0.910)	
**Laboratory model**	0.756 (0.633–0.865)	
**Combined clinical and laboratory model**	0.871 (0.776–0.947)	
**B. Paired comparisons of model discrimination**		
**Paired Comparison**	**ΔAUC (95% CI)**	***p*** **Value**
**Laboratory—Clinical**	−0.065 (−0.205 to 0.074)	0.362
**Combined—Clinical**	0.050 (0.005 to 0.101)	0.030
**Combined—Laboratory**	0.115 (0.005 to 0.232)	0.040

Abbreviations: AUC, area under the receiver operating characteristic curve; CI, confidence interval. Notes: AUCs were calculated from patient-level out-of-fold predictions generated by repeated nested cross-validation. Ninety-five percent confidence intervals for individual AUCs were estimated using 10,000 patient-level nonparametric bootstrap resamples. Differences in AUC between models were evaluated using paired patient-level bootstrap resampling of the corresponding out-of-fold predictions (10,000 resamples), with 95% confidence intervals derived from the bootstrap distribution. All *p* values were two-sided.

## Data Availability

The datasets generated and analysed during the current study are not publicly available due to patient privacy and confidentiality restrictions but are available from the corresponding author upon reasonable request.

## Supplementary Materials

The following supporting information can be downloaded at: https://www.mdpi.com/article/10.3390/jcm15187083/s1, Figure S1: Patient selection flow diagram; Figure S2: Calibration of the full-data combined model; Table S1. Available MRI characteristics of patients with spinal cord infarction and seronegative acute myelitis; Table S2. Treatment modalities in patients with spinal cord infarction; Table S3. Complete between-group comparisons and variable-level missingness for all 39 laboratory parameters; STROBE Statement—checklist of items that should be included in reports of observational studies; TRIPOD+AI 2024 Checklist [37].

## Abbreviations

The following abbreviations are used in this manuscript:

SAM	Seronegative acute myelitis
AQP4	Aquaporin-4
AUC	Area under the curve
BNP	B-type natriuretic peptide
CI	Confidence interval
CK	Creatine kinase
CK-MB	Creatine kinase myocardial band
CRP	C-reactive protein
CSF	Cerebrospinal fluid
DCA	Decision curve analysis
HDL-C	High-density lipoprotein cholesterol
IgG	Immunoglobulin G
IgA	Immunoglobulin A
IgM	Immunoglobulin M
INR	International normalized ratio
IQR	Interquartile range
IVIG	Intravenous immunoglobulin
LASSO	Least absolute shrinkage and selection operator
LDH	Lactate dehydrogenase
MRI	Magnetic resonance imaging
MOG	Myelin oligodendrocyte glycoprotein
OR	Odds ratio
ROC	Receiver operating characteristic
SCI	Spinal cord infarction
SD	Standard deviation
TnT	Troponin T
λ_1se_	Lambda one standard error
