# Supplementary material for: Routine Laboratory Parameters in the Differentiation of Spinal Cord Infarction and Seronegative Acute Myelitis: A Retrospective Study
Source: J Clin Med. 2026 Sep 12;15(18):7083. doi: 10.3390/jcm15187083 (PMC13607932; doi:10.3390/jcm15187083)
Supplement: Supplementary file 1 [file jcm-15-07083-s001.zip › STROBE_checklist.pdf]

STROBE Statement—checklist of items that should be included in reports of observational studies

	Item No.	Recommendation	Page No.	Relevant text from manuscript
**Title and abstract**	1	(*a*) Indicate the study's design with a commonly used term in the title or the abstract	1	The title identifies the report as a retrospective study.
		(*b*) Provide in the abstract an informative and balanced summary of what was done and what was found	1–2	The structured abstract reports the study design, setting, sample (34 SCI and 41 SAM), principal analyses, discrimination results, bootstrap internal validation, and conclusions.
**Introduction**				
Background/rationale	2	Explain the scientific background and rationale for the investigation being reported	2–4	The Introduction explains the diagnostic overlap between spontaneous SCI and SAM, the limitations of current diagnostic approaches, and the rationale for evaluating routinely available laboratory parameters.
Objectives	3	State specific objectives, including any prespecified hypotheses	4	Accordingly, we compared clinical characteristics and routinely measured laboratory parameters between spontaneous SCI and SAM and, as an exploratory analysis, developed and internally validated clinical, laboratory, and combined multivariable diagnostic models to evaluate whether routine laboratory

				parameters provided additional discriminatory information beyond selected clinical features.
**Methods**				
Study design	4	Present key elements of study design early in the paper	4	A retrospective single-center observational study was conducted.
Setting	5	Describe the setting, locations, and relevant dates, including periods of recruitment, exposure, follow-up, and data collection	4–5	The study was conducted at the Department of Neurology, Tianjin Medical University General Hospital, and included patients treated between January 2017 and June 2025. Laboratory samples were obtained within 48 hours of admission.
Participants	6	(*a*) *Cohort study*—Give the eligibility criteria, and the sources and methods of selection of participants. Describe methods of follow-up<br>*Case-control study*—Give the eligibility criteria, and the sources and methods of case ascertainment and control selection. Give the rationale for the choice of cases and controls<br>*Cross-sectional study*—Give the eligibility criteria, and the sources and methods of selection of participants	4	Separate operational diagnostic and exclusion criteria are provided for spontaneous SCI and SAM. Seventy potential SAM cases and 79 potential SCI cases were screened; 41 SAM and 34 SCI cases were included after independent multidisciplinary review.
		(*b*) *Cohort study*—For matched studies, give matching criteria and number of exposed and unexposed<br>*Case-control study*—For matched studies, give matching criteria and the number of controls per case	Not applicable	The study did not use matched participants.
Variables	7	Clearly define all outcomes, exposures, predictors, potential confounders, and effect modifiers. Give diagnostic criteria, if applicable	4–7, 12	Diagnostic criteria, demographic and clinical

				variables, the 39 blood laboratory parameters, the clinical, laboratory, and combined candidate predictor sets, outcome coding, and the final combined-model specification are described.
Data sources/ measurement	8*	For each variable of interest, give sources of data and details of methods of assessment (measurement). Describe comparability of assessment methods if there is more than one group	*4–6*	*Clinical information was extracted from medical records; radicular pain was coded from its documented presence or absence. Laboratory samples were collected within 48 hours of admission, and MRI findings were reviewed by experienced clinicians.*
Bias	9	Describe any efforts to address potential sources of bias	4, 6–7, 19	Potential bias was addressed through consensus diagnostic review, candidate predictors defined for the revised exploratory analysis, training-set-only preprocessing, repeated nested cross-validation, full-procedure bootstrap validation, and explicit discussion of selection, spectrum, and overfitting risks.
Study size	10	Explain how the study size was arrived at	4, 7	Seventy potential SAM cases and 79 potential SCI cases were screened; 41 SAM and 34 SCI cases were included. No formal

	sample-size calculation was performed because this was a retrospective cohort of all eligible patients.

Continued on next page

Quantitative variables	11	Explain how quantitative variables were handled in the analyses. If applicable, describe which groupings were chosen and why	5–6, 12	Continuous variables were summarized according to their distributions; all 39 laboratory parameters were reported as median (interquartile range). Predictors were standardized within training data. Hypertension and radicular pain were coded as binary variables.
Statistical methods	12	(*a*) Describe all statistical methods, including those used to control for confounding	5–7	The manuscript describes between-group tests, Benjamini–Hochberg false-discovery-rate correction, rank-biserial effect sizes, three penalized logistic models based on clinical, laboratory, and combined candidate sets, repeated nested cross-validation, bootstrap confidence intervals and paired model comparisons, full-procedure bootstrap internal validation, Brier scores, calibration, and decision-curve analysis.
		(*b*) Describe any methods used to examine subgroups and interactions	4, 15	The exploratory SCI treatment analysis was descriptive; no interaction or subgroup-effect model was fitted.
		(*c*) Explain how missing data were addressed	6	Predictor-specific missingness was quantified overall and by diagnostic group. Within each training set, missing values were replaced by training-set medians and the same

				values were applied to held-out data. S100 and BNP were excluded from model development because of substantial missingness.
		(*d*) *Cohort study*—If applicable, explain how loss to follow-up was addressed<br>*Case-control study*—If applicable, explain how matching of cases and controls was addressed<br>*Cross-sectional study*—If applicable, describe analytical methods taking account of sampling strategy	4	No longitudinal outcome or matched analysis was performed. Follow-up information was used for diagnostic reassessment when applying the eligibility criteria.
		(*e*) Describe any sensitivity analyses	6–7	No separate sensitivity analysis was conducted; robustness was evaluated using repeated nested cross-validation and bootstrap validation that repeated the complete model-development procedure.
**Results**				
Participants	13*	(a) Report numbers of individuals at each stage of study—eg numbers potentially eligible, examined for eligibility, confirmed eligible, included in the study, completing follow-up, and analysed	1, 4, 8	Seventy potential SAM cases and 79 potential SCI cases were screened. After exclusions, 41 SAM and 34 SCI participants were included and analyzed.
		(b) Give reasons for non-participation at each stage	4	For SAM, 29 of 70 potentially eligible cases were excluded: 12 did not fulfill the 2002 diagnostic criteria and 17 had an alternative diagnosis identified. For SCI, 45 of 79 potentially eligible cases were excluded: 37 were admitted more than seven days after symptom onset and 8 had a subsequent

				diagnostic revision during follow-up.
		(c) Consider use of a flow diagram	4	The participant screening and inclusion process is shown in Supplementary Figure S1.
Descriptive data	14*	(a) Give characteristics of study participants (eg demographic, clinical, social) and information on exposures and potential confounders	8–10	Baseline clinical characteristics and all laboratory results are reported for the 41 SAM and 34 SCI participants in Tables 1 and 2 and Supplementary Table S3.
		(b) Indicate number of participants with missing data for each variable of interest	6	The number and proportion of missing observations for each clinical and laboratory variable, overall and by diagnostic group, are reported in Supplementary Table S3.
		(c) *Cohort study*—Summarise follow-up time (eg, average and total amount)	Not applicable	The study did not summarize follow-up time because it was not a longitudinal outcome study.
Outcome data	15*	*Cohort study*—Report numbers of outcome events or summary measures over time	*7–15*	*Clinical and laboratory findings, diagnostic-model performance, internal validation, decision-curve analysis, and exploratory treatment findings are reported.*
		Case-control study—Report numbers in each exposure category, or summary measures of exposure		
		Cross-sectional study—Report numbers of outcome events or summary measures		
Main results	16	(*a*) Give unadjusted estimates and, if applicable, confounder-adjusted estimates and their precision (eg, 95% confidence interval). Make clear which confounders were adjusted for and why they were included	9–14	Table 2 reports laboratory medians, raw P values, false-discovery-rate-adjusted q values, and rank-biserial correlations. Table 3 reports AUCs and paired ΔAUC comparisons.

			Penalized coefficients, the final prediction formula, and bootstrap-validated performance are reported for the combined model.
	(*b*) Report category boundaries when continuous variables were categorized	5, 12	Continuous predictors were retained on their original continuous scales in the reported equation; hypertension and radicular pain were binary indicators.
	(*c*) If relevant, consider translating estimates of relative risk into absolute risk for a meaningful time period	Not applicable	No estimate was converted into a relative risk over a specified period.

Continued on next page

Other analyses	17	Report other analyses done—eg analyses of subgroups and interactions, and sensitivity analyses	11–15	Additional analyses include bootstrap predictor-selection frequencies, paired comparisons of model discrimination, the full-data penalized model equation, optimism correction, calibration and decision-curve analysis, single-marker ROC analyses, and exploratory treatment comparisons.
**Discussion**				
Key results	18	Summarise key results with reference to study objectives	15	The Discussion summarizes the principal clinical, laboratory, and model-development findings in relation to the study objectives.
Limitations	19	Discuss limitations of the study, taking into account sources of potential bias or imprecision. Discuss both direction and magnitude of any potential bias	19–20	Limitations include the retrospective single-center design, small sample size, possible diagnostic misclassification and spectrum bias, nonstandardized sampling relative to symptom onset and treatment, uncertain calibration, lack of external validation, incomplete imaging/CSF incremental-value assessment, and confounding in the exploratory treatment analysis.
Interpretation	20	Give a cautious overall interpretation of results considering objectives, limitations, multiplicity of analyses, results from similar studies, and other relevant evidence	15–19	The interpretation is cautious and considers the exploratory design, false-discovery-rate-adjusted comparisons, internal-validation results, limited sample size, and

				absence of external validation.
Generalisability	21	Discuss the generalisability (external validity) of the study results	19–20	The manuscript discusses the limited generalizability of findings from a single-center cohort and states that validation in larger, prospective, multicenter cohorts is required.
**Other information**				
Funding	22	Give the source of funding and the role of the funders for the present study and, if applicable, for the original study on which the present article is based	20	Funding sources and the role of the funders are reported in the Funding statement.

*Give information separately for cases and controls in case-control studies and, if applicable, for exposed and unexposed groups in cohort and cross-sectional studies.

Note: An Explanation and Elaboration article discusses each checklist item and gives methodological background and published examples of transparent reporting. The STROBE checklist is best used in conjunction with this article (freely available on the Web sites of PLoS Medicine at http://www.plosmedicine.org/, Annals of Internal Medicine at http://www.annals.org/, and Epidemiology at http://www.epidem.com/). Information on the STROBE Initiative is available at www.strobe-statement.org.
