# Supplementary material for: Routine Laboratory Parameters in the Differentiation of Spinal Cord Infarction and Seronegative Acute Myelitis: A Retrospective Study
Source: J Clin Med. 2026 Sep 12;15(18):7083. doi: 10.3390/jcm15187083 (PMC13607932; doi:10.3390/jcm15187083)
Supplement: Supplementary file 1 [file jcm-15-07083-s001.zip › Supplementary materials.pdf]

Supplementary Figure S1 Patient selection flow diagram. A total of 149 potentially eligible patients were screened, including 79 with possible spinal cord infarction (SCI) and 70 with possible seronegative acute myelitis (SAM). After applying the eligibility criteria, 34 patients with SCI and 41 patients with SAM were included in the final analysis.

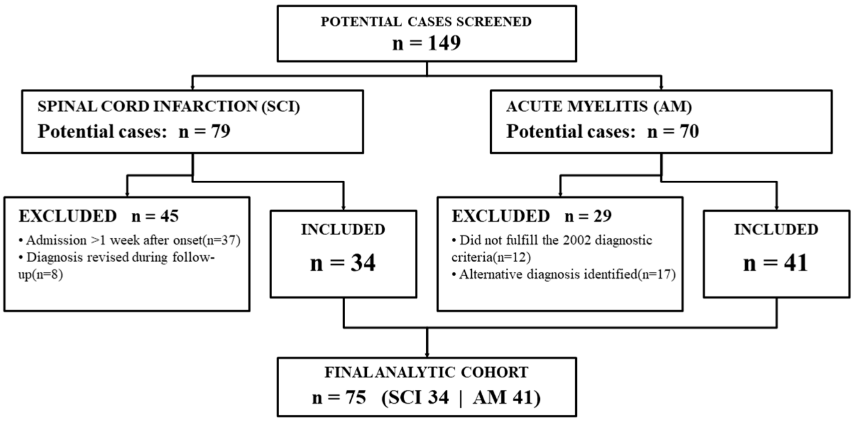

Supplementary Figure S2 Calibration of the full-data combined model. The dotted diagonal line represents ideal calibration, the gray dashed line represents the apparent calibration curve, and the solid black line represents the optimism-corrected calibration curve obtained from 1000 bootstrap resamples in which the complete model-development procedure was repeated. Open circles represent grouped observed event proportions, with vertical lines indicating 95% confidence intervals. The apparent calibration intercept and slope were 0.000 and 2.083, respectively; the optimism-corrected intercept was 0.039. The Brier score was 0.106 before and 0.129 after optimism correction. A scalar optimism-corrected calibration slope was not reported because its bootstrap estimate was numerically unstable. CI, confidence interval.

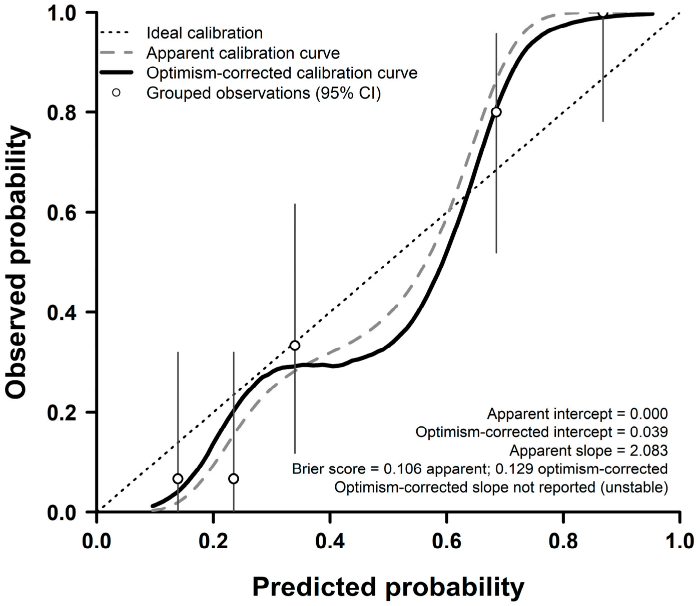

Supplementary Table S1. Available MRI characteristics of patients with spinal cord infarction and seronegative acute myelitis

MRI characteristic	SCI	SAM
**Onset-to-initial MRI interval, days**	1 (1–3), n=32	5 (1–12), n=41
**Lesion detected on initial MRI**	18/33 (54.5%)	30/41 (73.2%)
**Initially negative MRI**	15/33 (45.5%)	11/41 (26.8%)
**Lesion location**	**n=27**	**n=41**
**Cervical**	5/27 (18.5%)	12/41 (29.3%)
**Cervicothoracic**	3/27 (11.1%)	7/41 (17.1%)
**Thoracic**	14/27 (51.9%)	19/41 (46.3%)
**Thoracolumbar/conus**	4/27 (14.8%)	2/41 (4.9%)
**Other or unclear**	1/27 (3.7%)	1/41 (2.4%)
**Lesion length, vertebral segments**	4 (2–6), n=24	4 (2–7), n=38
**Lesion length ≥3 segments**	16/24 (66.7%)	28/38 (73.7%)
**Initial DWI positive**	1/33 (3.0%)	NA
**DWI positive on any available examination**	4/33 (12.1%)*	NA
**Anterior spinal artery territory pattern**	4/21 (19.0%)	NA
**Vertebral body signal abnormality**	7/33 (21.2%)	4/37 (10.8%)

Data are presented as median (interquartile range) or n/N (%). Denominators vary according to data availability. *One additional SCI patient had equivocal DWI findings. Among patients with initially negative MRI, one patient in each group had indeterminate follow-up imaging. Axial distribution, anterior versus posterior involvement, ADC findings, detailed T2 characteristics, contrast enhancement, and the owl-eye sign were not consistently documented. NA, not available; SCI, spinal cord infarction; SAM, Seronegative acute myelitis.

Table S2. Treatment modalities in patients with spinal cord infarction

Treatment	Total (n=34)	Motor improvement (⩾1 MRC grade) (n=12)	No motor improvement (<1 MRC grade) (n=22)
**Antiplatelet**	22 (64.7%)	6 (50.0%)	16 (72.7%)
**Anticoagulant**	20 (58.8%)	5 (41.7%)	15 (68.2%)
**Heparin**	14 (41.2%)	3 (25.0%)	11 (50.0%)
**Dehydrating agents**	14 (41.2%)	2 (16.7%)	12 (54.5%)
**Neurotropin**	30 (88.2%)	11 (91.7%)	19 (86.4%)
**Mecobalamin**	18 (52.9%)	8 (66.7%)	10 (45.5%)

**Corticosteroids/IVIG**	14 (41.2%)	3 (25.0%)	11 (50.0%)
**Volume expansion**	7 (20.6%)	2 (16.7%)	5 (22.7%)
**Lumbar drainage**	1 (2.9%)	0 (0%)	1 (4.5%)

Motor improvement was defined as an increase of ⩾1 MRC grade from admission to discharge.

Supplementary Table S3. Complete between-group comparisons and variable-level missingness for all 39 laboratory parameters

Laboratory parameter	Unit	SAM available / missing, n (%)	SAM, median (IQR)	SCI available / missing, n (%)	SCI, median (IQR)	Unadjusted P value	BH-adjusted q value	Rank-biserial correlation
Complement C3	mg/dL	37 / 4 (9.8%)	92.2 (83.4–103.0)	31 / 3 (8.8%)	98.1 (91.4–116.0)	0.100	0.260	0.234
Complement C4	mg/dL	37 / 4 (9.8%)	23.7 (19.6–26.6)	31 / 3 (8.8%)	25.3 (20.8–29.5)	0.141	0.290	0.209
C-reactive protein	mg/L	37 / 4 (9.8%)	0.30 (0.17–0.45)	31 / 3 (8.8%)	0.84 (0.49–3.34)	<0.001	**<0.001**	0.614
Immunoglobulin G	mg/dL	37 / 4 (9.8%)	1090.0 (972.0–1200.0)	31 / 3 (8.8%)	1140.0 (975.0–1340.0)	0.554	0.642	0.085
Immunoglobulin A	mg/dL	37 / 4 (9.8%)	221.0 (181.0–301.0)	31 / 3 (8.8%)	217.0 (170.0–325.5)	0.834	0.879	−0.031
Immunoglobulin M	mg/dL	37 / 4 (9.8%)	101.0 (69.0–150.0)	31 / 3 (8.8%)	73.9 (60.4–97.4)	0.020	0.071	−0.330
Thrombin time	s	36 / 5 (12.2%)	19.4 (18.5–21.0)	33 / 1 (2.9%)	18.9 (17.4–20.2)	0.319	0.497	−0.141
D-dimer	ng/mL	37 / 4 (9.8%)	358.0 (267.0–787.0)	34 / 0 (0.0%)	617.5 (351.8–1268.5)	0.127	0.282	0.211
Fibrinogen	g/L	37 / 4 (9.8%)	3.00 (2.57–3.29)	34 / 0 (0.0%)	3.34 (2.79–4.31)	0.009	0.052	0.359
International normalized ratio	—	37 / 4 (9.8%)	1.09 (1.00–1.13)	34 / 0 (0.0%)	1.02 (0.96–1.07)	0.199	0.337	−0.178
B-type natriuretic peptide	pg/mL	33 / 8 (19.5%)	12.5 (5.6–26.9)	24 / 10 (29.4%)	40.2 (9.5–176.0)	0.009	0.052	0.408

Troponin T	μg/L	33 / 8 (19.5%)	0.007 (0.004–0.011)	28 / 6 (17.6%)	0.013 (0.006–0.024)	0.012	0.059	0.373
S100	ng/L	5 / 36 (87.8%)	87.5 (79.0–91.9)	4 / 30 (88.2%)	74.4 (72.7–75.3)	0.178	0.315	−0.600
Total cholesterol	mmol/L	35 / 6 (14.6%)	4.54 (4.06–5.28)	33 / 1 (2.9%)	4.82 (4.16–5.49)	0.731	0.792	0.049
Triglycerides	mmol/L	35 / 6 (14.6%)	1.18 (0.85–1.62)	33 / 1 (2.9%)	1.65 (1.34–2.40)	0.002	**0.025**	0.438
HDL cholesterol	mmol/L	34 / 7 (17.1%)	1.08 (0.97–1.26)	33 / 1 (2.9%)	0.90 (0.81–1.07)	0.006	**0.049**	−0.389
LDL cholesterol	mmol/L	34 / 7 (17.1%)	2.62 (2.22–3.26)	33 / 1 (2.9%)	2.82 (2.31–3.49)	0.482	0.627	0.101
Total protein	g/L	37 / 4 (9.8%)	66.0 (64.0–69.0)	33 / 1 (2.9%)	65.0 (61.0–70.0)	0.625	0.696	−0.069
Albumin	g/L	37 / 4 (9.8%)	39 (37–42)	33 / 1 (2.9%)	37 (34–40)	0.039	0.118	−0.286
Alanine aminotransferase	U/L	38 / 3 (7.3%)	17.0 (11.2–27.8)	33 / 1 (2.9%)	19.0 (14.0–27.0)	0.560	0.642	0.081
Aspartate aminotransferase	U/L	37 / 4 (9.8%)	17.0 (13.0–20.0)	33 / 1 (2.9%)	18.0 (15.0–23.0)	0.243	0.394	0.163
Creatinine	μmol/L	36 / 5 (12.2%)	55.5 (49.8–69.5)	31 / 3 (8.8%)	66.0 (55.0–88.0)	0.018	0.071	0.336
Creatine kinase	U/L	35 / 6 (14.6%)	79.0 (48.0–105.0)	30 / 4 (11.8%)	63.5 (48.5–93.0)	0.396	0.532	−0.124
Creatine kinase-MB	U/L	35 / 6 (14.6%)	8.0 (5.5–11.5)	30 / 4 (11.8%)	9.5 (7.0–13.0)	0.387	0.532	0.126
White blood cell count	$\times 10^9$/L	41 / 0 (0.0%)	7.62 (6.03–9.54)	34 / 0 (0.0%)	7.81 (6.64–8.88)	0.534	0.642	0.085
Red blood cell count	$\times 10^{12}$/L	41 / 0 (0.0%)	4.40 (4.13–4.62)	34 / 0 (0.0%)	4.58 (4.29–4.92)	0.119	0.282	0.211
Hemoglobin	g/L	41 / 0 (0.0%)	133.0 (126.0–144.0)	34 / 0 (0.0%)	140.0 (130.5–147.8)	0.168	0.315	0.187
Platelet count	$\times 10^9$/L	41 / 0 (0.0%)	229.0 (194.0–265.0)	34 / 0 (0.0%)	199.5 (181.2–235.0)	0.067	0.187	−0.247
Neutrophil percentage	%	41 / 0 (0.0%)	72.3 (65.1–78.8)	34 / 0 (0.0%)	69.8 (60.9–75.1)	0.354	0.532	−0.126
Lymphocyte	%	41 / 0 (0.0%)	20.3 (15.4–26.2)	34 / 0 (0.0%)	19.6 (14.6–26.2)	0.962	0.962	−0.007

percentage								
Monocyte percentage	%	40 / 1 (2.4%)	6.45 (5.63–7.50)	34 / 0 (0.0%)	7.70 (6.83–9.43)	0.001	**0.022**	0.442
Eosinophil percentage	%	40 / 1 (2.4%)	0.70 (0.00–1.40)	34 / 0 (0.0%)	1.20 (0.53–2.00)	0.031	0.102	0.290
Basophil percentage	%	40 / 1 (2.4%)	0.30 (0.18–0.43)	34 / 0 (0.0%)	0.30 (0.20–0.50)	0.378	0.532	0.118
Absolute neutrophil count	$\times 10^9$/L	41 / 0 (0.0%)	5.51 (3.72–7.22)	34 / 0 (0.0%)	5.54 (4.35–6.76)	0.932	0.957	0.012
Absolute lymphocyte count	$\times 10^9$/L	40 / 1 (2.4%)	1.52 (1.14–1.90)	34 / 0 (0.0%)	1.64 (1.15–2.12)	0.519	0.642	0.088
Absolute monocyte count	$\times 10^9$/L	40 / 1 (2.4%)	0.45 (0.36–0.62)	34 / 0 (0.0%)	0.62 (0.49–0.78)	0.005	**0.044**	0.385
Absolute eosinophil count	$\times 10^9$/L	40 / 1 (2.4%)	0.05 (0.00–0.11)	34 / 0 (0.0%)	0.10 (0.03–0.16)	0.015	0.064	0.329
Absolute basophil count	$\times 10^9$/L	40 / 1 (2.4%)	0.020 (0.010–0.030)	34 / 0 (0.0%)	0.020 (0.020–0.040)	0.171	0.315	0.181
Hematocrit	%	40 / 1 (2.4%)	40.0 (37.5–42.0)	34 / 0 (0.0%)	41.5 (38.4–44.6)	0.130	0.282	0.206

Notes: Values are presented as median (interquartile range). Available/missing columns report the number of nonmissing observations and the number (percentage) of missing observations within each diagnostic group (SAM, n=41; SCI, n=34). Between-group comparisons used two-sided Mann–Whitney U tests. P values were adjusted across all 39 laboratory parameters using the Benjamini–Hochberg false-discovery-rate procedure. Positive rank-biserial correlations indicate higher values in the SCI group. The S100 comparison is based on only nine available observations and should be interpreted cautiously. SAM, Seronegative acute myelitis; BH, Benjamini–Hochberg; BNP, B-type natriuretic peptide; CRP, C-reactive protein; HDL, high-density lipoprotein; IQR, interquartile range; LDL, low-density lipoprotein; SCI, spinal cord infarction.
