# Supplementary material for: Routine Laboratory Parameters in the Differentiation of Spinal Cord Infarction and Seronegative Acute Myelitis: A Retrospective Study
Source: J Clin Med. 2026 Sep 12;15(18):7083. doi: 10.3390/jcm15187083 (PMC13607932; doi:10.3390/jcm15187083)
Supplement: Supplementary file 1 [file jcm-15-07083-s001.zip › TRIPOD.pdf]

TRIPOD+AI 2024 Checklist

Item No.	Recommendation	Page No.	Relevant text from manuscript
**TITLE**			
1	Identify the study as developing or evaluating the performance of a multivariable prediction model, the target population, and the outcome to be predicted.	1	Title: ‘Routine Laboratory Parameters in the Differentiation of Spinal Cord Infarction and Seronegative Acute Myelitis: A Retrospective Study.’ The title identifies the population and diagnostic outcome but does not explicitly identify prediction-model development.
**ABSTRACT**			
2	See the TRIPOD+AI for Abstracts checklist.	1–2	The abstract reports the retrospective design, 75 participants (34 SCI and 41 AM), candidate predictor sets, penalized logistic regression, repeated nested cross-validation, bootstrap internal validation, AUC estimates, and the exploratory decision-curve analysis. Model availability and calibration results are not reported in the abstract.
**INTRODUCTION**			
3a	Explain the healthcare context (including whether diagnostic or prognostic) and rationale for developing or evaluating the prediction model, including references to existing models.	3–4	‘In clinical practice, spontaneous SCI is frequently misdiagnosed.’ ‘Identifying objective and widely accessible biomarkers that can help differentiate SCI from inflammatory causes would facilitate earlier and more accurate diagnosis, and may help avoid unnecessary or potentially harmful treatments.’
3b	Describe the target population and the intended purpose of the prediction model in the context of the care pathway, including its intended users (e.g., healthcare professionals, patients, public).	4 (partial)	‘Accordingly, we compared clinical characteristics and routinely measured laboratory parameters between spontaneous SCI and seronegative AM and evaluated whether these parameters could complement clinical and MRI-based differentiation.’ Intended users and the precise point of use in the care pathway are not explicitly specified.
3c	Describe any known health inequalities between sociodemographic groups.	Not reported	Not reported in the current manuscript.
4	Specify the study objectives, including whether the study describes the development or validation of a prediction model (or both).	4	‘Accordingly, we compared clinical characteristics and routinely measured laboratory parameters between spontaneous SCI and seronegative AM and evaluated whether these parameters could complement clinical and MRI-based differentiation.’ The manuscript describes model development with internal validation.
**METHODS**			
5a	Describe the sources of data separately for the development and evaluation datasets (e.g., randomised trial, cohort, routine care or registry data), the rationale for using these data, and representativeness of the data.	4 (partial)	‘We conducted a retrospective analysis of patients diagnosed with acute spontaneous spinal cord infarction or acute myelitis at the Department of Neurology, Tianjin Medical University General Hospital…’ Data representativeness is not explicitly discussed in Methods.
5b	Specify the dates of the collected participant data, including start and end of participant accrual; and, if applicable, end of follow-up.	4	‘…between January 2017 and June 2025.’ Follow-up data were available for 30 patients; the study is diagnostic, so a prediction time horizon is not applicable.
6a	Specify key elements of the study setting (e.g., primary care, secondary care, general population), including the number and location of centres.	4	Patients were identified at the Department of Neurology, Tianjin Medical University General Hospital; the study is described as single-centre in the Discussion.
6b	Describe the eligibility criteria for study participants.	4	SCI required acute spinal-cord-attributable neurological deficits, onset within 72 hours, compatible MRI findings, and exclusion of other causes. AM cases were reassessed using the 2002 Transverse Myelitis Consortium Working Group criteria; alternative causes, NMOSD, and MOGAD were excluded.
6c	Give details of any treatments received, and how they were handled during model development or evaluation, if relevant.	5	‘Because of the retrospective design, the timing of blood sampling relative to acute treatments, including corticosteroids, intravenous immunoglobulin, antithrombotic therapy, and intravenous fluids, could not be determined consistently for all patients.’ Treatments were not model predictors.
7	Describe any data pre-processing and quality checking, including whether this was similar across relevant sociodemographic groups.	6 (partial)	‘Within each training set, missing predictor values were replaced with the corresponding training-set medians, and predictors were standardized using the training-set means and standard deviations. These preprocessing parameters were then applied unchanged to the corresponding held-out data.’ Comparability across sociodemographic groups is not reported.

Item No.	Recommendation	Page No.	Relevant text from manuscript
8a	Clearly define the outcome that is being predicted and the time horizon, including how and when assessed, the rationale for choosing this outcome, and whether the method of outcome assessment is consistent across sociodemographic groups.	4 (partial)	SCI was coded as 1 and seronegative AM as 0. Diagnostic criteria and case ascertainment are described. A prognostic time horizon is not applicable; consistency across sociodemographic groups is not reported.
8b	If outcome assessment requires subjective interpretation, describe the qualifications and demographic characteristics of the outcome assessors.	4 (partial)	'Two experienced neurologists and a neuroradiologist independently reviewed all cases, and any discrepancies were resolved by consensus.' Assessor demographic characteristics are not reported.
8c	Report any actions to blind assessment of the outcome to be predicted.	Not reported	No blinding of diagnostic outcome assessment to candidate predictors is described.
9a	Describe the choice of initial predictors (e.g., literature, previous models, all available predictors) and any pre-selection of predictors before model building.	6	The clinical, laboratory, and combined candidate sets are listed. 'Candidate variables were defined according to clinical relevance, routine availability, data completeness, and avoidance of redundant measures rather than univariate statistical significance. B-type natriuretic peptide was excluded because 24% of observations were missing.'
9b	Clearly define all predictors, including how and when they were measured (and any actions to blind assessment of predictors for the outcome and other predictors).	5–6 (partial)	Demographic, clinical, and laboratory predictors are listed; routine blood samples were collected after an overnight fast on the morning following admission, and laboratory units are reported in Tables 2 and 3. Blinding of predictor assessment is not reported.
9c	If predictor measurement requires subjective interpretation, describe the qualifications and demographic characteristics of the predictor assessors.	Not reported / not applicable	Most laboratory predictors were objectively measured. Qualifications and demographic characteristics of assessors for clinical predictors such as radicular pain are not reported.
10	Explain how the study size was arrived at (separately for development and evaluation), and justify that the study size was sufficient to answer the research question. Include details of any sample size calculation.	4–6 (partial)	All eligible retrospective cases were included (75 participants; 34 SCI events). The manuscript acknowledges that the limited number of events created a risk of overfitting, but no formal sample-size calculation or explicit sufficiency justification is provided.
11	Describe how missing data were handled. Provide reasons for omitting any data.	6; Supplementary Table S3	Before imputation, the number and proportion of missing observations were quantified for each clinical and laboratory variable, overall and separately in the SCI and AM groups. Missing predictor values were replaced with training-set medians, which were then applied unchanged to held-out data. BNP was omitted from candidate modeling because 24% of observations were missing. Supplementary Table S3 reports variable-specific available and missing counts and percentages for all 39 laboratory parameters in each diagnostic group.
12a	Describe how the data were used (e.g., for development and evaluation of model performance) in the analysis, including whether the data were partitioned, considering any sample size requirements.	7	Discrimination was estimated using 50 repetitions of nested stratified five-fold cross-validation. Preprocessing, penalty selection, and fitting were confined to outer training folds, with predictions generated for held-out folds and averaged per participant.
12b	Depending on the type of model, describe how predictors were handled in the analyses (functional form, rescaling, transformation, or any standardisation).	6	Within each training set, missing values were replaced by training medians and predictors were standardized using training-set means and standard deviations; these values were applied unchanged to held-out data.
12c	Specify the type of model, rationale, all model-building steps, including any hyperparameter tuning, and method for internal validation.	6–7	LASSO logistic regression was applied to three prespecified predictor sets. Ten-fold cross-validation and the one-standard-error rule were used for penalty selection. Repeated nested five-fold cross-validation and 1,000 full-procedure bootstrap refits were used for internal validation; preprocessing, tuning, selection, and penalized fitting were repeated within resamples.
12d	Describe if and how any heterogeneity in estimates of model parameter values and model performance was handled and quantified across clusters (e.g., hospitals, countries).	Not applicable	Single-centre study; there were no multiple centres or prespecified clusters across which heterogeneity could be quantified.
12e	Specify all measures and plots used (and their rationale) to evaluate model performance (e.g., discrimination, calibration, clinical utility) and, if relevant, to compare multiple models.	7	AUC with 95% CI, paired ΔAUC, Brier score, calibration intercept and slope, calibration plot, predictor-selection frequency, and decision-curve net benefit were assessed. DCA was explicitly designated exploratory because no clinical intervention or decision threshold was prespecified.
12f	Describe any model updating (e.g., recalibration) arising from the model evaluation, either overall or for particular sociodemographic groups or settings.	Not applicable	No external model evaluation or model updating was performed.

Item No.	Recommendation	Page No.	Relevant text from manuscript
12g	For model evaluation, describe how the model predictions were calculated (e.g., formula, code, object, application programming interface).	Not applicable	This was a model-development study with internal validation rather than an external evaluation of an existing model.
13	If class imbalance methods were used, state why and how this was done, and any subsequent methods to recalibrate the model or the model predictions.	Not applicable	No over-sampling, under-sampling, SMOTE, or other class-imbalance correction was reported; the groups comprised 34 SCI and 41 AM participants.
14	Describe any approaches that were used to address model fairness and their rationale.	Not reported	No formal fairness analysis or subgroup performance evaluation is reported; the manuscript notes that the small sample precluded robust subgroup analyses.
15	Specify the output of the prediction model (e.g., probabilities, classification). Provide details and rationale for any classification and how the thresholds were identified.	7	The model output was a predicted probability of SCI. No risk categories or fixed classification threshold were defined. DCA examined threshold probabilities from 0.01 to 0.80 and was considered exploratory.
16	Identify any differences between the development and evaluation data in healthcare setting, eligibility criteria, outcome, and predictors.	Not applicable	No independent evaluation dataset was used; internal validation used resampling of the same single-centre cohort.
17	Name the institutional research board or ethics committee that approved the study and describe participant-informed consent or the ethics committee waiver of informed consent.	Methods/Declarations (page number pending final pagination)	The study was conducted in accordance with the Declaration of Helsinki and was approved by the Ethics Committee of Tianjin Medical University General Hospital on 26 February 2026 (ID: IRB2026-YX-068-01). Written informed consent was obtained from all participants or their legal guardians prior to inclusion in the study.
**OPEN SCIENCE**			
18a	Give the source of funding and the role of the funders for the present study.	Not reported	Funding source and funder role are not reported in the current manuscript.
18b	Declare any conflicts of interest and financial disclosures for all authors.	Declarations (page number pending final pagination)	The authors declare no conflicts of interest.
18c	Indicate where the study protocol can be accessed or state that a protocol was not prepared.	Not reported	Protocol availability, or a statement that no protocol was prepared, is not reported.
18d	Provide registration information for the study, including register name and registration number, or state that the study was not registered.	Not reported	Study registration, or a statement that the study was not registered, is not reported.
18e	Provide details of the availability of the study data.	Declarations (page number pending final pagination)	The individual-level raw data are not publicly available because their release is restricted by the study ethics approval and participant privacy requirements.
18f	Provide details of the availability of the analytical code.	7 (partial)	Analyses were performed using SPSS 26.0 and R 4.1.2 with pROC, rms, glmnet, and rmda. Package versions, licences, computing environment, and code availability are not reported.
**PATIENT AND PUBLIC INVOLVEMENT**			
19	Provide details of any patient and public involvement during the design, conduct, reporting, interpretation, or dissemination of the study, or state no involvement.	Not reported	Patient and public involvement, or a statement that there was no involvement, is not reported.
**RESULTS**			

Item No.	Recommendation	Page No.	Relevant text from manuscript
20a	Describe the flow of participants through the study, including the number of participants with and without the outcome and, if applicable, a summary of the follow-up time. A diagram may be helpful.	4; Supplementary Figure S1	The analysis included 75 participants (34 SCI and 41 AM). Seventy-nine potential SCI cases were screened; patients admitted more than seven days after symptom onset and those subsequently reclassified during follow-up were excluded, leaving 34 SCI cases. Seventy potential AM cases were reassessed; 29 were excluded, leaving 41 AM cases. The complete selection process is shown in Supplementary Figure S1.
20b	Report the characteristics overall and, where applicable, for each data source or setting, including key dates, key predictors, treatments received, sample size, number of outcome events, follow-up time, and amount of missing data. Report differences across key demographic groups.	7–12; Supplementary Table S3 (partial)	Table 1 reports demographic and clinical characteristics for 41 AM and 34 SCI participants. Table 2 reports the laboratory parameters with nominal P<0.05. Supplementary Table S3 provides complete distributions, available sample sizes, and group-specific missing counts and percentages for all 39 laboratory parameters. Performance across demographic subgroups was not estimated because of the limited sample size.
20c	For model evaluation, show a comparison with the development data of the distribution of important predictors (demographics, predictors, and outcome).	Not applicable	No independent external evaluation dataset was used.
21	Specify the number of participants and outcome events in each analysis (e.g., for model development, hyperparameter tuning, model evaluation).	1–2, 7, 10	The analysis included 75 participants (34 SCI events and 41 AM controls). The same cohort was used in repeated nested cross-validation and 1,000 bootstrap resamples. Fold-level counts are not reported.
22	Provide details of the full prediction model (e.g., formula, code, object, API) to allow predictions in new individuals and to enable third-party evaluation and implementation, including any restrictions to access or re-use.	Results, Model specification (verify page after final pagination)	The full-data combined penalized model is reported with the selected value of lambda, intercept, all seven nonzero coefficients, and the probability transformation. The linear predictor is: −2.937179 + 0.018072×age + 0.417311×hypertension + 2.052281×radicular pain + 0.122950×CRP + 0.187146×triglycerides + 0.482027×absolute monocyte count + 2.594441×absolute eosinophil count. The predicted probability of SCI is 1/[1+exp(−linear predictor)]. Access or reuse restrictions for the analytical code remain to be stated under Item 18f.
23a	Report model performance estimates with confidence intervals, including for any key subgroups (e.g., sociodemographic). Consider plots to aid presentation.	10–14 (partial)	Repeated nested cross-validation AUCs with 95% CIs are reported for the clinical, laboratory, and combined models, together with paired DeltaAUC estimates. For the full-data combined model, the apparent and optimism-corrected AUCs are 0.937 and 0.902, and the corresponding Brier scores are 0.106 and 0.129. The apparent calibration intercept and slope are 0.000 and 2.083; the optimism-corrected intercept is 0.039. A scalar optimism-corrected slope is not reported because its bootstrap estimate was numerically unstable; an optimism-corrected calibration curve is provided. Subgroup performance was not estimated.
23b	If examined, report results of any heterogeneity in model performance across clusters.	Not applicable	No cluster-level heterogeneity analysis was performed in this single-centre study.
24	Report the results from any model updating, including the updated model and subsequent performance.	Not applicable	No model updating following an independent model evaluation was performed.
**DISCUSSION**			
25	Give an overall interpretation of the main results, including issues of fairness, in the context of the objectives and previous studies.	15–19 (partial)	The Discussion interprets clinical and laboratory findings, distinguishes marginal association from multivariable prediction, and compares findings with prior biological literature. Formal fairness analyses were not performed and fairness is not discussed.
26	Discuss any limitations of the study (such as a non-representative sample, sample size, overfitting, missing data) and their effects on bias, statistical uncertainty, and generalizability.	19	Limitations include the retrospective single-centre design, selection bias, unmeasured confounding, 34 SCI events, possible misclassification and spectrum bias, non-standardized sampling and pretreatment, lack of external validation, incomplete calibration assessment, and inability to conduct robust subgroup analyses.
27a	Describe how poor-quality or unavailable input data (e.g., predictor values) should be assessed and handled when implementing the prediction model.	Not reported	The manuscript describes median imputation during model development but does not specify how missing or poor-quality input values should be handled at clinical implementation.
27b	Discuss whether users will be required to interact in handling the input data or using the model, and what level of expertise is required of users.	Not reported	User interaction, training, and expertise requirements for model implementation are not described.
27c	Discuss any next steps for future research, with a specific view to applicability and generalizability of the model.	19	‘Prospective multicenter studies should validate the model, assess calibration, and determine its incremental value alongside standardized MRI and CSF evaluation.’

Source: Collins GS, Moons KGM, Dhiman P, et al. TRIPOD+AI statement: updated guidance for reporting clinical prediction models that use regression or machine learning methods. BMJ. 2024;385:e078378. doi:10.1136/bmj-2023-078378. Checklist version: 7 February 2024.
